# Familiarity Perception Call Elicited under Restricted Sensory Cues in Peer-Social Interactions of the Domestic Chick

**DOI:** 10.1371/journal.pone.0058847

**Published:** 2013-03-08

**Authors:** Mamiko Koshiba, Yuka Shirakawa, Koki Mimura, Aya Senoo, Genta Karino, Shun Nakamura

**Affiliations:** 1 Tokyo University of Agriculture and Technology, Tokyo, Japan; 2 National Center of Neurology and Psychiatry, NCNP, Tokyo, Japan; Utrecht University, The Netherlands

## Abstract

Social cognitive mechanisms are central to understanding developmental abnormalities, such as autistic spectrum disorder. Peer relations besides parent-infant or pair-bonding interactions are pivotal social relationships that are especially well developed in humans. Cognition of familiarity forms the basis of peer socialization. Domestic chick (Gallus gallus) studies have contributed to our understanding of the developmental process in sensory-motor cognition but many processes remain unknown. In this report, we used chicks, as they are precocial birds, and we could therefore focus on peer interaction without having to consider parenting. The subject chick behavior towards familiar and unfamiliar reference peers was video-recorded, where the subject and the reference were separated by either an opaque or transparent wall. Spectrogram and behavior correlation analyses based on principal component analysis, revealed that chicks elicited an intermediate contact call and a morphologically different distress call, more frequently towards familiar versus unfamiliar chicks in acoustic only conditions. When both visual and acoustic cues were present, subject chicks exhibited approaching and floor pecking behavior, while eliciting joyful (pleasant) calls, irrespective of whether reference peers were familiar or unfamiliar. Our result showed that chicks recognized familiarity using acoustic cues and expressed cognition through modified distress calls. These finding suggests that peer affiliation may be established by acoustic recognition, independent of visual face recognition, and that eventually, both forms of recognition are integrated, with modulation of acoustic recognition.

## Introduction

Neurobiological understanding of socio-emotional cognition is crucial to diagnosis and therapeutic intervention in social-domain specific developmental disorders, such as autistic spectrum disorders and other psychiatric illnesses. Recent advances in behavioral and neurobiological studies on neonatal development of sensory-motor cognition in animates and specification of social individuals [1] have revealed domain-relevant biases toward faces [1],[2] and this type of attention bias is not restricted to face recognition, but also towards self-propelled causal agency [3] and biological motion recognition [4]–[7]. Similar arguments can be put forward in relation to auditory [1], [8] and olfactory recognition [9].

Domestic chick (Gallus gallus) studies have contributed to the formulation of a two process theory, namely CONSPEC and CONLERN. CONSPEC proposes that widely divergent vertebrates possess similar domain-relevant biases toward faces. A chick's natural predisposition mechanisms adhere to CONSPEC. Additionally, attention biases lead to neuronal based development of species specific individual cognition or CONLERN. It is thought that chick, imprinting mechanisms correlate to CONLERN, and are processed through the intermediate medial mesopallium (IMM) area [1]. The neuronal substrates relevant to CONSPEC and CONLERN comprise the subcortical face-recognition route, which provides a developmental foundation for what later becomes the adult cortical ‘social brain’ network [10]. However, it is uncertain how early neuronal substrates for social affiliation are integrated as part of the social brain network, a process that is crucial to understanding socio-emotional development and its disorders. Here, we developed a peer social affiliation chick model, covering a series of developmental stages, and focused on familiarity cognition using acoustic cues.

In animal communication behavior, the “call” occupies a unique position, since it is a direct sound transmission of the sender's emotional state [11]. The receiver can then decode the sound and make a response in the form of an action or another call. This mutual interaction makes a communication loop, allowing both the sender and receiver to understand the meaning of calls [12]. Thus, it is crucial to understand and investigate call behavior in the context of socio-ecological interaction [13]. Contact calls have been studied extensively and the individuality of mate and kin relationships in mammals and birds has been recognized [13]. To date, there are few reports describing familiarity recognition within con-specific or hetero-specific groups, beyond mate and kin relationships [14],[15]. These observations suggest that some types of contact calls are learned and can dynamically change structure during social interactions [16],[17]. However, it is uncertain how the sensory cues of the social communicator are related to the changes in vocal structure. Since the pioneering study of chick calls by Collias & Joos [18], spectrograph analysis has revealed great detail in call types, along with the behavioral and functional relevance of each call. Domestic fowl chicks emit four different types of calls, a distress call (cheeps, peeps), pleasure notes (twitterings), an intermediate call (short peep), and a fear trill [18].

In this study, two groups of chicks were reared separately and call behavior was examined in interaction tests observing reactions between familiar and unfamiliar chicks. All animals were provided with acoustic cues but at times were deprived of visual cues. To assess call function, we performed multivariate analysis based on principal component analysis and visualized the correlation structure of call types and other behavior parameters [19], such as floor pecking. When restricted to acoustic cues, subject chicks emitted intermediate calls more often to familiar chicks, relative to unfamiliar chicks, as well as a complex distress call. We found no significant differences in subject chick call behavior when both visual and acoustic cues were present in the meetings with familiar and unfamiliar reference chicks.

## Results

### 1. Call modulation mediated by repeated meetings with familiar reference chicks

**Figure 1:**
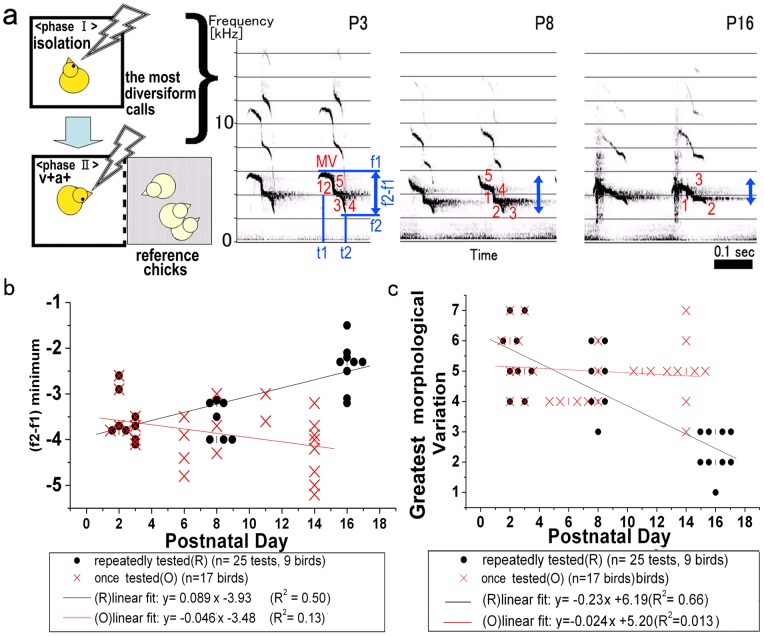
Age- or repetition-dependent call modulation. a. Subject chick behavior was video recorded in the serial social context depicted in the left scheme (isolation and v−a+ contact). Spectrograms on the right showed typical calls produced by one subject chick on postnatal days 3, 8 and 16 (P3, 8, 16). The subject was exposed to the same reference chicks at P3, P8, and P16. Typical morphological values are indicated by serial numbers (1–5 at P3, 1–5 at P8 and 1–3 at P16). Calls emitted at P3 and P8 were defined as a “D-call”, and calls at P16 as a “dj-call”. The call type and morphology are described in Materials and Methods. b. Modulation of call frequency differences (f2−f1 value) over developmental time. The average of five minimum values of f2−f1 (negative values, since frequency f1>f2) during behavioral tests was plotted against the test day (postnatal day). Black dots represent daily values of subject chicks meeting the same reference chicks during a test, against post natal day of development (the repeatedly-tested chicks), and red-crosses signify values of subject chicks meeting unfamiliar chicks during a test against post natal day of development (once tested chicks). The colored lines represent the respective linear regression fitted line. c. Modulation of call morphology over developmental time. The black dots represent values from repeatedly-tested chicks (see above) and red-crosses, values from once tested chicks (see above). The colored lines represent the respective linear regression fitted line.

First, we examined call development in subject chicks, over postnatal days 3 to 16 (P3 to P16) (Figure 1a). The spectrogram showed a decline in the frequency difference in kHz (f2−f1) and morphological variation (MV) of the first call-component. This experiment design, shown in Figure 1a, consisted of two contexts. In Phase I, the subject chick was placed in isolation and in Phase II, the subject chick was exposed visually and acoustically to the same reference chicks over time. Our results suggested that call modulation in subject chicks may be a natural result of development, as well as an adaptation to repeated exposure to the same reference chicks in the meeting test. Next, we examined this assumption by comparing the call frequency difference (f2−f1) between a subject chick exposed repeatedly to the same reference chicks (repeatedly tested, R) and a subject chick exposed once to unfamiliar chicks (once-tested, O) (Figure 1b). The regression line fitting of the O-group chick was slightly negative over time, although the Pearson's correlation coefficient was low (R2 = 0.13). On the other hand, the regression line (black) slope of the R-group chick increased (R2 = 0.50) over time, suggesting an effect for repetitive exposure to familiar chicks. Next, we compared MV values between the two test groups (Figure 1c). The linear fit line slope of the O-group remained almost constant over time (R2 = 0.013), while that of the R-group decreased significantly (R2 = 0.67). Taken together, these results implied that repeated meetings with familiar chicks modulated call behavior in subject chicks, hinting at the existence of social memory. The next step was to investigate the possible relationship between call morphology and social experience, by examining first f2−f1 and then MV parameters.

### 2. Social context dependent shifts in call types, towards familiar and unfamiliar reference chicks

**Figure 2:**
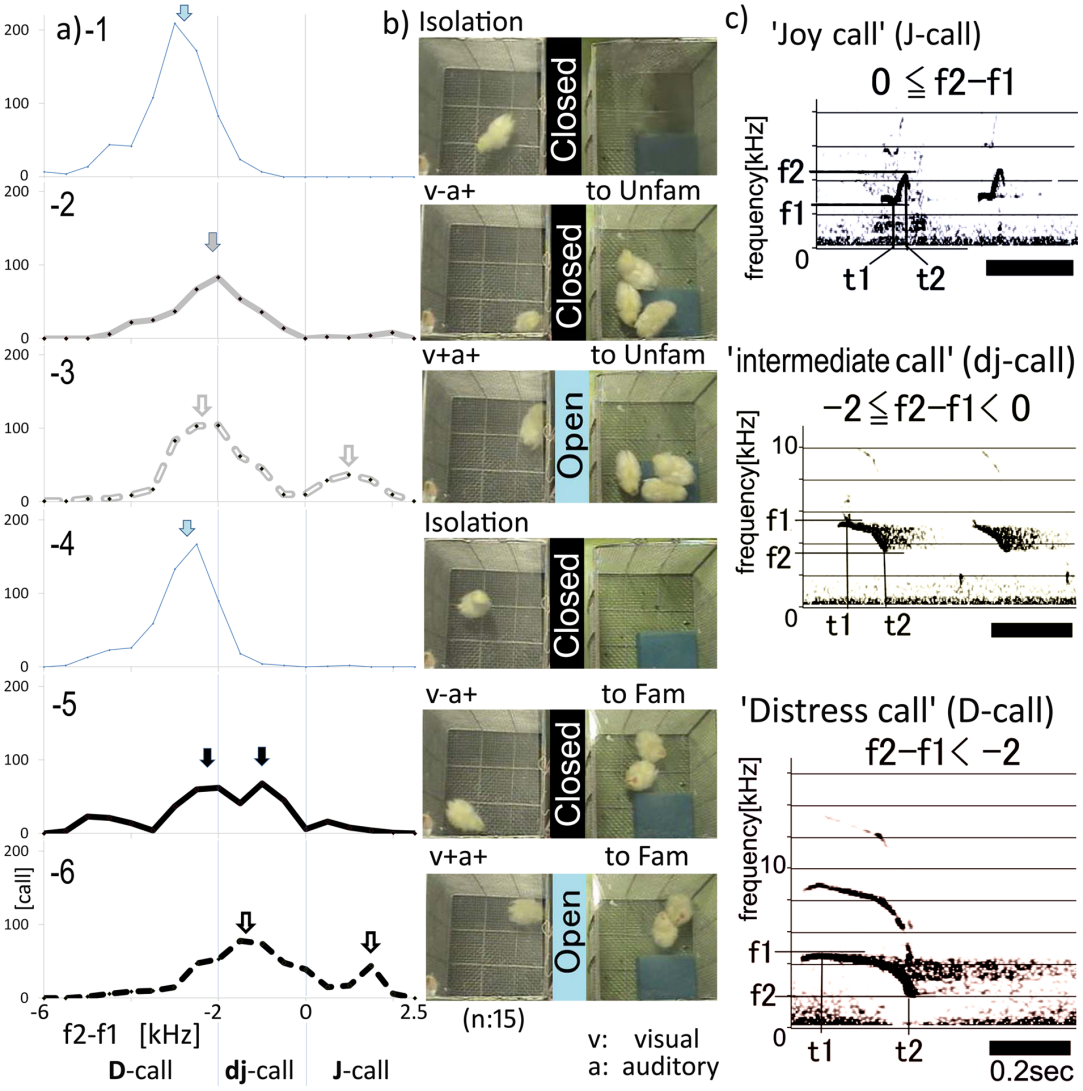
Changes of call type defined by f2−f1 frequency in different social contexts. a. Shifting distribution of f2−f1frequency in different social contexts. The series a−1 to a−6 correspond to the social contexts described in Figure 2b. The peak call number at particular f2−f1 values (kHz) is marked by arrows in each social context. Details are described in the text. b. The serial context is as follows: first, isolation; second, acoustic only exposure to unfamiliar reference chicks (v−a+); third, visual and acoustic exposure (v+a+) to unfamiliar reference chicks; fourth, a second period of isolation; fifth, v−a+ exposure to familiar chicks and sixth, v+a+ exposure to familiar reference chicks. c. Typical spectrogram of subject chick calls. D, dj and J, denote the D-call, dj-call and J-call respectively. The D-call is a “negative” expression signifying “dissatisfaction” or “distress”, the J-call is a “positive expression” signifying something “pleasant” or “joyful”, while the dj call is an intermediate call between the D and J calls. The black bar denotes 0.2 seconds.

**Figure 3:**
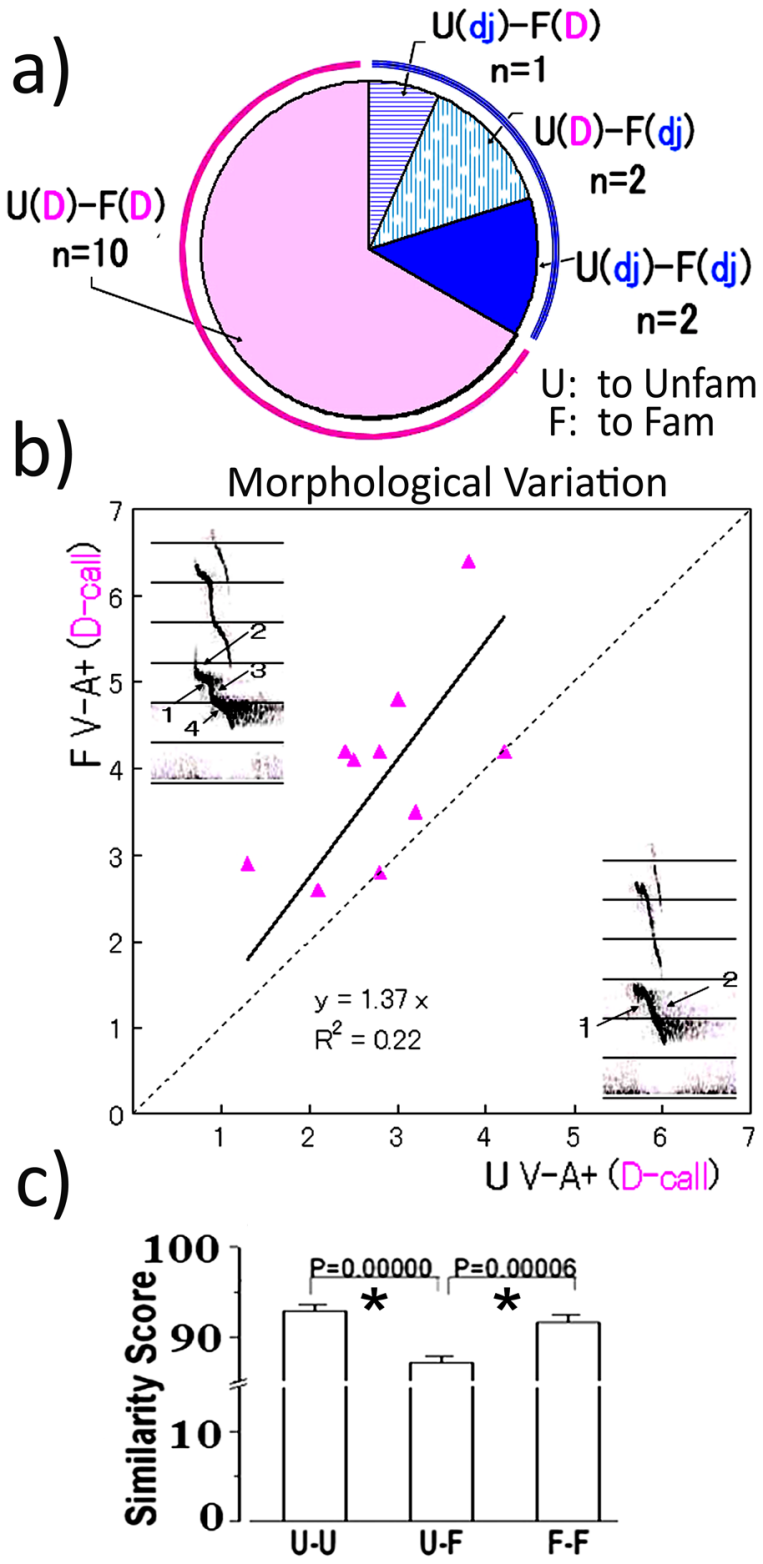
Difference in the morphological variation of D calls with familiarity. a. Call type combinations from subject chicks to unfamiliar (U) and familiar (F) reference chicks. Each subject chick met an unfamiliar or familiar reference chick during this test. We counted calls from subject chicks for 30 seconds after an initial call from reference chicks. Only D- and dj-calls were observed in the v−a+ context. U(D)–F(dj) denotes meetings where the test chick emitted D-call including dj-call to unfamiliar chicks and the same chick emitted dj-calls without D-calls to familiar chicks b. The correlation plot of the call number against familiar and unfamiliar chicks in the acoustic only context. The morphological variation of U(D) calls was plotted against F(D) calls in the V−A+ context (n = 10). Here, the call frequency was higher towards familiar reference chicks. c. Comparison of D calls emitted to familiar and unfamiliar reference chicks. Each call was analyzed by Sound Analysis pro using three pairs; U(D)–U(D), U(D)–F(D) and F(D)–F(D). The error bar denotes the standard error of mean. The similarity score for U(D)–F(D) calls was significantly lower than for the other pairings by One-Way ANOVA, and Two sample independent t-test. In this analysis, we compared calls from pairs of U–U (10 pairs per session), U–F (25 pairs per session), and F–F (10 pairs per session) meetings.

**Figure 4:**
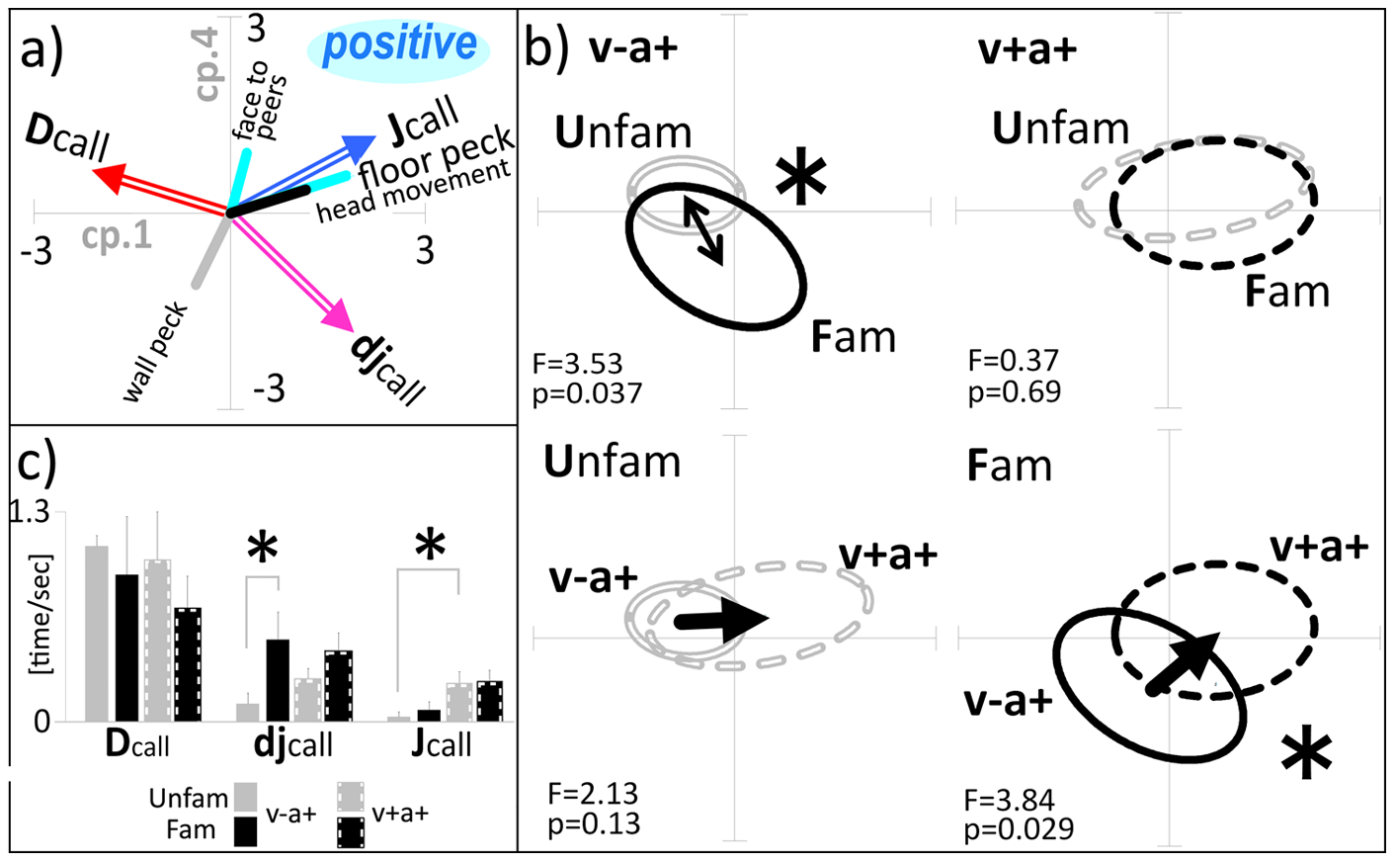
Integrated analysis of social behavior towards familiar and unfamiliar reference peers by PCA. a. Subject behavior was video recorded in the v−a+ and v+a+ contexts. Seven factors were selected to differentiate responses in behavior towards familiar and unfamiliar peers after the Two sample independent t-test, and multivariate analysis by correlation matrix-based PCA (see detailed description of behavior parameters used for PCA in the text). b. The scores from the 1st and 4th component by independent PCA for v−a+ (left-upper) and v+a+ (right-upper) were approximated, with each variance ellipse by variance, co-variance-based PCA for social behavior towards familiar (Fam: black line or dotted) and unfamiliar (Unfam: grey out line or dotted) peers. The factor loading positive vectors drawn from the averaged center were adjusted three fold. Clear differences between Fam and Unfam could be seen, as Fam values distributed lower on the 4th component in the v−a+ but not v+a+ context. The direction of Fam specific distribution in the v−a+ context could be explained by the following factor loading vectors (see Figure 4a and text). The statistical significance between Fam and Unfam behavior was evaluated by Wilks' lambda (see Materials and Methods). c. Call frequency of test chick towards familiar or unfamiliar reference chicks in the v−a+ and v+a+ contexts. P-values were calculated by two-sample T-test and the asterisks show significant values. Call frequency of dj calls was significantly higher towards familiar reference chicks only in the v−a+ context. However, J call frequency in the v−a+ context significantly increased relative to calls in the v+a+ context but only towards unfamiliar reference chicks.

A subject chick met both familiar (Figure 2b, Fam) and unfamiliar (Figure 2b, Unfam) chicks in a series of tests (Figure 2b). The series consisted of Familiar and Unfamiliar meetings within three contexts (isolation, acoustic only cues (v−a+), and visual and acoustic cues (v+a+), (see more details in Materials and Methods). To avoid a possible bias in the meeting order, we randomized the order in which subject chicks met Fam and Unfam reference chicks. First, we classified three call types according to the frequency difference (f2−f1) within the first component of the call sonogram (Figure 2c). From 15 birds, we plotted 30-second periods of data in a call-number histogram, with f2−f1 values from −6 to 2.5[kHz], and with 0.5 kHz bin steps in each context (Figure 2a). In the isolation context (Figures 2a–1 and –4), a single peak appeared at around −3[kHz], irrespective of whether the call was to Fam or Unfam chicks. In the (v−a+) context, twin peaks appeared in calls to Fam chicks, but a single peak appeared in calls to Unfam chicks (Figures 2a–2 and –5). On the other hand, in the (v+a+) context, the difference between calls to Fam and Unfam chicks was more subtle (Figures 2a–3 and –6). These results suggested that the dj-call may convey information of familiarity in the v−a+ context. More detailed analysis of call frequency in each context is described below.

### 3. Modulation of call morphological variation depending on the recognition of familiarity

We introduced call morphological variation (MV) as a parameter which may correlate with the cognition of familiarity in Figure 1. We further assessed this point by comparing MV scores of subject chicks calls to Fam and to Unfam chicks in the v−a+ context. Since MV scores appeared higher in D- rather than dj- or J-calls, we compared MV scores for D-calls elicited by reference chicks. Each subject chick randomly met both Fam and Unfam peers in separate tests. We then classified the subject call type elicited for 30 seconds, in response to a call from the reference chick. From the 15 subject chicks, we noted four combinations of call types as summarized in Figure 3a. A plot of MV values from 10 subject D-calls emitted in response to Unfam (U) and Fam (F) chick calls is shown in Figure 3b. A liner fit line showed MV values toward familiar peers was significantly higher than toward unfamiliar peers. To examine this result further, we compared morphological similarity, using sound analysis software distributed by Dr. O. Tchernichovski [20]. The similarity scores of U–F were significantly lower than those for U–U and F–F (Figure 3c), confirming a morphological difference between D-calls to Fam and Unfam peers.

### 4. Transient dj-calls as a parameter of familiarity recognition

Subject chick behavior changed depending on the social context. Typically, chicks froze or roved, emitting a D-call when in isolation, roved and pecked the surrounding walls emitting a D- or dj-call in an acoustic only context, or approached peers, pecking the floor and emitting a J-call in the visual and acoustic cue context (see more details in Materials and Methods). In this analysis, we aimed to identify behavioral parameters that differ quantitatively in each context by defining the complex correlation of the three call types and the behavioral parameters, using principal component analysis (PCA). The social contexts examined were U v−a+ , U v+a+, F v−a+ and F v+a+, where the capital letter denotes a meeting with either Unfamiliar (U) or Familiar (F) reference peers and the lower case letters denote the context of either acoustic stimulus only (v−a+) or acoustic and visual stimuli (v+a+). The behavioral parameters were extracted from video data, as follows; floor-peck, wall-peck, face to peers, and head-movement (see more details in Materials and Methods). The correlation between behavioral parameters and D-, dj- and J-call frequency was then investigated by PCA with correlation matrices, and visualized as factor loading vectors (Figure 4a). The most significant difference in behavioral features between Unfam and Fam peer meetings appeared in the v−a+ context (F = 3.53, p = 0.037, Wilks' lambda), in contrast to the v+a+ context (Figure 4b, upper panel). The parameters contributing to differences can be explained by the factor loading vectors. The distribution of the behavioral vector in the Fam context after PCA (Figure 4b), expanded into the 4th quadrant and correlated positively with the dj-call factor loading vector and negatively with the D-call vector in the v−a+ context. Two sampled t-tests between Unfam and Fam call behavior showed a significant difference in dj-call frequency (Figure 4c, djcall). On the other hand, there was no significant difference between meetings with Unfam and Fam peers in the v+a+ context (Wilks' lambda; F = 0.37, p = 0.69). Next, we compared context dependent changes of behavior in Unfam and Fam meetings (Figure 4b bottom panel). Only in the Fam series, was the context-dependent behavior shift (from v−a+ to v+a+) statistically significant (Wilks' lambda; F = 3.84, P = 0.029), primarily due to the shift in J-call and floor-pecking parameters in their positive directions, however J-call was significantly different between v−a+ and v+a+ in Unfam (Figure 4c). In summary, we found that dj-call frequency correlated with familiarity cognition in the v−a+ context and the high frequency of J calls as well as floor pecking in the context shift from v−a+ to the v+a+ irrespective of whether reference peers were familiar or unfamiliar.

Matrix-based PCA suggested similar modulations from the v−a+ to v+a+ contexts, with increasing J-calls and floor pecking behavior, as positive factors, and with no behaviors related to D- or dj-calls (Figure 4). To represent the structure of the most correlated multiple parameters within either the v−a+ or v+a+ contexts, each context was examined by PCA and then the factor loadings were represented as positively directed vectors from the averaged center. Furthermore, to visualize the specific areas representing subject chicks toward familiar or unfamiliar peers, the ellipse variance approximation was superimposed. This resulted in a greater difference between the F and U meetings in the v−a+ context. This difference appeared not in the 1st, but the 4th component (unpaired two sample T-test for unequal sample sizes and unequal variance in the 4th component: P = 0.0095). In contrast, the subjects showed very similar behavior towards familiar and unfamiliar chicks in the v+a+ context. These combined results suggested that dj-call was a unique behavior elicited from subject chicks toward familiar peers in the v−a+ context. It may be related to Morton's motivation-structure rules [21] that call type shift from D-call to dj-call during isolation to v−a+ and dj-call to j-call during v−a+ to v+a+.

## Discussion

In this study, we found that the recognition of familiarity was expressed in three ways by 15 day old subject chicks. Firstly, the rate of dj-calls in the v−a+ context increased when emitted to familiar peers relative to unfamiliar peers. Secondly, D-call morphology showed greater complexity in calls emitted to familiar peers in the v−a+ context. Lastly, the rate of J-calls increased when emitted to familiar peers, with concomitant approach and floor pecking behavior in the v+a+ context from the v−a+ context. This result suggested that acoustic cues of familiar peers elicited dj-calls as well as D-call complexity and that visual cues elicited J-calls irrespective of familiar or unfamiliar perception. Chicks were deprived of the somatosensory cues resulting from pecking and floor scratching, by placing the boxes containing a subject and reference peers on separate platforms. Similarly the sensory cue of olfaction was also removed or at least diminished by covering the test box with a transparent plastic sheet, to prevent air flow between the cages. These combined results suggested that dj-call was a unique behavior elicited from subject chicks toward familiar peers in the v−a+ context. It may be related to Morton's motivation-structure rules [21] that call type shift from D-call to dj-call during isolation to v−a+ and dj-call to j-call during v−a+ to v+a+. Our finding suggests that peer affiliation can be established by acoustic recognition, independent of visual face recognition [1]–[2] and when integrated, acoustic recognition is modulated. Widely divergent vertebrates possess similar domain-relevant biases toward visual facial cues and similar arguments have been made for auditory cues [1] [8] [22]. However, the precise mechanisms for visual recognition dominance are undetermined, The mechanisms relating to how cross modal sensory integration develops at behavioral and neurobiological levels, is a subject for further research.

Subject chicks met reference chicks either through being reared in the same cage from hatching (familiar reference) or reared in a different group (unfamiliar reference). In the familiar reference tests, we observed a decreased call rate and intensity (f2−f1 value), even though the subject and reference chicks had shared the same cage until the day of testing. The reason for this call modulation in the repeated familiar reference test may be related to the fact that in unfamiliar cages, subject chicks suffered stress, as indicated by roving behavior and an accompanying D-call. In this situation, meeting familiar peers may reduce this stress, while unfamiliar peers may impose additional stress [8] [23],[24]. As repeated testing affected call behavior, we used a single test to evaluate chick behavior towards familiar and unfamiliar references. As mentioned earlier, the highly dynamic nature of the contact call has already been recognized and the development of individual chick recognition proceeds in parallel with the segregation of inter group call perception [25]–[27]. Chicks recognize a number of maternal calls, including the food call, follow me call, roosting call, predator call, and fear call [24],[28]–[30]. It is unknown if chicks are capable of recognizing familiar peer calls based on individual chick recognition. In this study, we did not address the issue of whether subject chicks recognized the calls of individual reference chicks in familiarity cognition, nor did we examine the type of call that induced calls expressing familiarity. Since our test situation presented reference chicks in groups of two to four chicks, a different type of study at a future date is needed examine these intriguing questions.

The dj-call was the major call type elicited to familiar reference chicks in the v−a+ context. The intermediate call has not been as well characterized in literature as the J-call (pleasure note, twittering call) or D-call (distress call, peeps). Marx et al., [31] showed that intermediate calls (short peeps) were elicited during a step-wise isolation paradigm, in a particular starting group size. Call types in the last step, where subject chicks were left alone, differed significantly depending on the initial peer group size. If the group size was greater than four, the major call type emitted was a D-call, with no intermediate calls. Where subject chicks started the step-wise isolation test in groups of two or three, intermediate and D-calls were emitted. The interpretation of this behavior is currently uncertain. Considering that dj-calls are emitted from chicks in step-wise isolation and independent of group size, this call may be related to search attention [11] and alert conditions. Electrical stimulation of the intercollicular nucleus (ICo) in the mesencephalon, induced distress calls in control chicks, and crowing in testosterone-treated chicks [32]. However, it is unclear whether D- and dj-calls are variants, derived from the same call output center, or whether there is a specified motor control center for each call, which is regulated differentially by emotional and cognitive neural networks [33]. Studies examining immediate early gene expression, and multi-point in vivo recording and micro-dialysis using awake animals, should shed light on these important questions [11],[34],[35].

In this study, integrative analysis of multi-behavioral parameters was effective in identifying the characteristic structure of complex expressions, such as social behavior, even though the analysis is based solely on the extraction of behavioral parameters from video recordings, without any assumption of inter-parameter correlation. It is feasible that PCA could be applied to the objective translation of social non-verbal communication and/or non-social interaction with the environment by animals, including humans. This method may well provide support to intra and inter-species communication studies, as an information processing interface.

## Materials and Methods

### 1. Animals

This experimental protocol was approved by the Ethics review Committee for Animal.

Experiments of the National Institute of Neuroscience, NCNP (18–40) and Tokyo University of Agriculture and Technology, TUAT (19–19). These committees follow the animal care and experimental guidelines of Japan Neuroscience Society and NIH, in USA.

Fertilized eggs from domestic chicks (Gallus gallus domestics), White Leghorn, Maria strain, were purchased from a local breeder, Miyake Fukajo (Chiba, Japan). They were kept in a dark incubator (Showa Furanki) at 37.7 degrees centigrade with approximately 50% humidity and automatic rolling every hour. On embryonic day 21 (E21, the start of incubation was defined as E 1), which usually coincided with one day before hatching (the day of hatching was defined as post natal day 1, P1), three or four eggs were moved to incubator-boxes in different rooms, separated by thick concrete walls and ceilings, thus minimizing communication between the two sets of birds.

Each incubator box was kept at around 28 degrees centigrade, with a light bulb (10 watt). Constant lighting was maintained from E21 until P3, after which a 12 h dark-light cycle was set. We used three different sized incubator boxes ((width) × (depth) × (height) in cm: 20×20×20, 35×32×33 and 52×40×53) to evaluate the effects of box size on the development of affiliation behavior. The peers reared in the same incubator were denoted as familiar peers (Fam or F), while those from different incubators were denoted as unfamiliar peers (Unfam or U).

During rearing, we avoided social interaction with chicks, since handling can induce stress or affiliation effects on chicks [13], [36]. Chicks were transferred using a small opaque container during daily incubator cleaning.

### 2. Behavioral Tests and the definition of typical behavior

Behavioral tests were conducted in the same room where the subject birds (7 males and 6 females, age: average-standard deviation. 12.7–3.1 postnatal day) were reared. The subject chick was put into one of the two transparent metal net boxes covered with plastic sheets (29 (w) ×29 (d) ×29 (h) cm) and the reference chicks into another. The two boxes were placed adjacent to each other, separated only by a masking board that was either removed manually or electrochromically controlled. The box containing the subject chick was covered with a transparent acrylic board, in order to reduce olfactory cues from the reference bird and researchers, and also to increase the specificity of call recordings from subject birds through a microphone in the test box.

As seen in Figures 2a–1, subject chicks underwent the following six serial peer-social contexts: context 1; initial isolation period with no reference chicks, and a masking board in place, context 2; Unfamiliar or Familiar chicks (U/F) were presented with acoustic only cues, ensured by a separation board (v−a+) , context 3; U/F chicks were presented with both visual and acoustic cues, after removing the separation board (v+a+), context 4; second period of isolation, context 5; similar to context 2, but chicks met alternative peers, that is F or U chicks, and context 6; similar to context 3, but the order of chick presentation was reversed (that is, F or U). Each context lasted for 1–2 minutes. All behavior was recorded using a top video camera (SONY handycam) with an external microphone in the test box. The recorded WMV files were transferred into WAVE and JPEG files using TMPGEnc-2.5 software (Pegasys Inc., Tokyo). The subject's typical behavior in each context was defined according to the following eight kinds of typical behavior:- 1; Freeze with no call, 2; Freeze with D-call, 3; Freeze with dj-call, 4; Roving with D-call, 5; Roving with dj-call, 6; Roving with J-call, 7; Approach, and 8; G-move. The g-move was defined in this study as approaching behavior, with the chick moving back and forth along the separation board in the v+a+ context, thought to represent grouping behavior. In the parameters of Figure 4a, “face to peers” defined as beak angle to adjacent cage within 45 degrees (beak-to-separating wall) refers to subject chick's preferred head position towards the reference chicks. We further defined pecking behavior, pecking floor (floor peck) and pecking cage wall (wall peck) expressed as frequency of pecking behavior per specified time.

### 3. Call analysis

We used free software (Syrinx, version 2.4i) distributed by Dr. John Burt (University of Washington) to analyze chick calls. Spectrograms were used to identify call types by analyzing morphology of the first component (Figure 1 to Figure 3). This analysis sometimes allowed us to detect unique morphological features from all call components. We defined three call types based on the frequency change of the first component over time, as follows: First, we read the first time point (Figure 1a, t1) where the frequency was maximum/minimum (f1), and the second time point (t2) where the frequency minimum/maximum (f2). Next, we calculated Δ(delta)f  =  (f2−f1). Finally, if Δ(delta)f <−2, we designated this call as a D-call. If Δ(delta)f >0, as a J-call, and if −2< Δ(delta)f <0, as a dj-call. A JPEG file was made of all call spectrograms and the frequency difference Δ(delta)f (f2−f1) was measured using Image J software (NIH, USA). Morphological complexity of the 1st component was measured by counting the number of negative curvatures moving anti-clockwise along the edge of the morphological Figure, keeping the Figure on the left side. The call data from each context covered the duration times as shown below:

1. Context 1 and 4, first or second isolation period, respectively; 0–30 sec.
2. Context 2, U or F under V−A+ condition; for 30 sec after the 1st U/F J- or dj-call.
3. Context 3, U or F under V+A+ condition; for 30 sec after the 1st U/F J- or dj-call.

We also confirmed call similarity by Sound Analysis Pro Software [20].

### 4. Statistics

Statistical analysis was performed using Excel (Microsoft) and OriginPro ver 7.5 (OriginLab). Linear regression was calculated using Pearson correlation coefficient.

### 5. Multi-behavioral parameter assays using principal component analysis

In order to integrate multi-behavioral parameters, we used principal component analysis (PCA) using the free software, MLVAR95 based on a correlation matrix (http://home.a02.itscom.net/coffee/takoindex.html). In some cases, results were confirmed by manual calculations using Excel (Microsoft, Office 2003). The calculated scores were plotted in a two dimensional (2D) plane defined by PCA components. To compare behavioral patterns among the different groups, we applied the second PCA to each group of data and fitted the covariance as an ellipse, where the long axis was derived from the 1st component and the short axis from the 2nd component of sub-PCA, using the variance-covariance matrix. The following 18 parameters were averaged over the test duration: 1; velocity of head-center movement (head movement), 2; direction of head movement, 3–6; local preference (time ratio) at four partial areas, 7; face to peers, 8–10; frequency of D-, dj- and J-calls [time/sec], 11–13; sonogram detected numbers of D-, dj- and J-calls, 14; duration ratio of Freeze with D-call, 15; duration ratio of Freeze with no call; 16–18; duration of pecking at wall (wall peck), floor (floor peck) or self. The effective parametric combination for PCA was screened to visualize variance ellipses with the greatest differences relating to response behavior between F and U chicks, by identifying unique areas. In the end, the seven parameters, 1, 7, 11–13, 16 and 18 (see above) were used in the final analysis and PCA plane was screened to focus on difference between Fam and Unfam. The statistical analysis after PCA was performed by Wilks' lambda [19].

## References

[1] Morton J, Johnson MH (1991) CONSPEC and CONLERN: A Two-Process Theory of Infant Face Recognition. Psychological Review 98: 164–181. 2047512 10.1037/0033-295x.98.2.164

[2] Salva OR, Farroni T, Regolin L, Vallortigara G (2011) The Evolution of Social Orienting: Evidence from Chicks (Gallus gallus) and Human Newborns. PLoS ONE 6: e18802. 21533093 10.1371/journal.pone.0018802 PMC3080385

[3] Mascalzonia E, Regolina L, Vallortigara G (2010) Innate sensitivity for self-propelled causal agency in newly hatched chicks. PNAS 107: 4483–4485. 20160095 10.1073/pnas.0908792107 PMC2840119

[4] Vallortigara G, Regolin L, Marconato F (2005) Visually Inexperienced Chicks Exhibit Spontaneous Preference for Biological Motion Patterns. PLoS Biology 3: e208. 15934787 10.1371/journal.pbio.0030208 PMC1150290

[5] Simion F, Regolin L, Bulf H (2008) A predisposition for biological motion in the newborn baby. PNAS 105: 809–813. 18174333 10.1073/pnas.0707021105 PMC2206618

[6] Klin A, Lin DJ, Gorrindo P, Ramsay G, Jones W (2009) Two-year-olds with autism orient to nonsocial contingencies rather than biological motion. Nature 459: 257–261. 19329996 10.1038/nature07868 PMC2758571

[7] Brown J, Kaplan G, Rogers LJ, Vallortigara G (2010) Perception of biological motion in common marmosets (Callithrix jacchus): by females only. Anim Cogn 13: 555–564. 20052512 10.1007/s10071-009-0306-0

[8] Vallortigara G (1988) Chicks in a Novel Environment: Effects of Conspecific Calls. Ethology 78: 241–345.

[9] Vallortigara G, Andrew RJ (1994) Olfactory lateralization in the chick. Neuropsychologia 32: 417–423. 8047249 10.1016/0028-3932(94)90087-6

[10] Johnson MH (2005) Subcortical face processing. Nat Rev Neurosci 6: 766–774. 16276354 10.1038/nrn1766

[11] Andrew RJ (1969) Intracranial self-stimulation in the chick and the causation of emotional behavior. Annals New York Academy of Sciences 159: 625–639. 10.1111/j.1749-6632.1969.tb12967.x 5260292

[12] MacKay DM (1972) Formal analysis of communicative processes. In: Hinde RA eds. Non-verbal communication. Cambridge: Cambridge University Press. pp. 3–25.

[13] Kondo N, Watanabe S (2009) Contact calls: Information and social function. Japanese Psychological Research 51: 197–208.

[14] Braune P, Schmidt S, Zimmermann E (2008) Acoustic divergence in the communication of cryptic species of nocturnal primates (Microcebus ssp.). BMC Biol 6: 19. 18462484 10.1186/1741-7007-6-19 PMC2390514

[15] Boughman JW (1998) Vocal learning by greater spear-nosed bats. Proc Biol Sci 265: 227–233. 9493408 10.1098/rspb.1998.0286 PMC1688873

[16] Lemasson A, Ouattara K, Petit EJ, Zuberbuhler K (2011) Social learning of vocal structure in a nonhuman primate? BMC Evol Biol 11: 362. 22177339 10.1186/1471-2148-11-362 PMC3260242

[17] Kondo N, Izawa E, Watanabe S (2012) Crows cross-modally recognize group members but not non-group members. Proc Biol Sci 279: 1937–1942. 22217722 10.1098/rspb.2011.2419 PMC3311902

[18] Collias N, Joos M (1953) The Spectrographic analysis of sound signals of the domestic fowl. Behavior 5: 175–188.

[19] Koshiba M, Mimura K, Sugiura Y, Okuya T, Senoo A, et al (2011) Reading marmoset behavior ‘semantics’ under particular social context by multi-parameters correlation analysis. Prog Neuropsychopharmacol Biol Psychiatry 35: 1499–1504. 21300127 10.1016/j.pnpbp.2011.01.021

[20] Tchernichovski O, Lints TJ, Deregnaucourt S, Cimenser A, Mitra PP (2004) Studying the song development process: rationale and methods. Ann N Y Acad Sci 1016: 348–363. 15313784 10.1196/annals.1298.031

[21] Morton ES (1977) On the occurrence and significance of motivation-structural rules in some bird and mammal sounds. The American Naturalist 111: 855–869.

[22] Muir DW, Abraham W, Forbes B, Harris LS (1979) The ontogenesis of an auditory localization response from birth to four months of age. Canadian Journal of Psychology 33: 320–333. 546497 10.1037/h0081729

[23] Feltenstein MW, Sufka KJ (2005) Screening antidepressants in the chick separation-stress paradigm. Psychopharmacology (Berl) 181: 153–159. 15778882 10.1007/s00213-005-2227-1

[24] Kikusui T, Winslow JT, Mori Y (2006) Social buffering: relief from stress and anxiety. Philos Trans R Soc Lond B Biol Sci 361: 2215–2228. 17118934 10.1098/rstb.2006.1941 PMC1764848

[25] Bouchet H, Blois-Heulin C, Pellier AS, Zuberbühler K, Lemasson A (2012) Acoustic variability and individual distinctiveness in the vocal repertoire of red-capped mangabeys (Cercocebus torquatus). J Comp Psychol 126: 45–56. 21875177 10.1037/a0025018

[26] Konishi M (1963) The role of auditory feedback in the vocal behavior of the domestic fowl. Zeitschrift fur Tierpsychologie 20: 349–367. 5874921

[27] Field SE, Rickard NS, Toukhsati SR, Gibbs ME (2007) Maternal hen calls modulate memory formation in the day-old chick: the role of noradrenaline. Neurobiol Learn Mem 88: 321–330. 17507256 10.1016/j.nlm.2007.04.001

[28] Bolhuis JJ (1999) Early learning and the development of filial preferences in the chick. Behav Brain Res 98: 245–252. 10683113 10.1016/s0166-4328(98)00090-4

[29] Ellis JM, Riters LV (2012) Vocal parameters that indicate threat level correlate with FOS immunolabeling in social and vocal control brain regions. Brain Behav Evol 79: 128–140. 22179056 10.1159/000334078 PMC3355646

[30] Kent JP (1989) On the Acoustic Basis of Recognition of the Mother Hen by the Chick in the Domestic-Fowl (Gallus-Gallus). Behaviour 108: 1–9.

[31] Marx G, Leppelt J, Ellendorff F (2001) Vocalisation in chicks (Gallus gallus dom.) during stepwise social isolation. Applied Animal Behaviour Science 75: 61–74.

[32] Yazaki Y, Matsushima T, Aoki K (1999) Testosterone modulates stimulation-induced calling behavior in Japanese quails. J Comp Physiol A 184: 13–19. 10077861 10.1007/s003590050302

[33] Panksepp J (2011) Cross-species affective neuroscience decoding of the primal affective experiences of humans and related animals. PlosOne 6: e21236. 10.1371/journal.pone.0021236 PMC3168430 21915252

[34] Kosfeld M, Heinrichs M, Zak PJ, Fischbacher U, Fehr E (2005) Oxytocin increases trust in humans. Nature 435: 673–676. 15931222 10.1038/nature03701

[35] Ellis JM, Riters LV (2012) Vocal parameters that indicate threat level correlate with FOS immunolabeling in social and vocal control brain regions. Brain Behav Evol 79: 128–140. 22179056 10.1159/000334078 PMC3355646

[36] Bolhuis JJ (1999) Development of perceptual mechanisms in birds: predispositions and imprinting. In: Bolhuis JJ, Hogan JA eds. The development of animal behavior, A reader. Oxford: Blackwell Publishers Ltd. pp. 176–191.

